# Braïdism

**Published:** 1860-10-01

**Authors:** 


### Art. V.—BRAIDISM.*

A HOPE has long been indulged by enthusiastic experimenters, that artificially induced somnambulism might be applied to some good purpose in the treatment of disease. When, in this country, Mr. Braid, by his ingenious researches, first threw a clear light upon the etiology of artificial somnambulism, he did not overlook the possible therapeutic value of the experimental method which he had adopted in his analysis of the facts of so-called "animal magnetism,"—a method which led to the discovery of that series of somnambulic phenomena which were designated by him, and which have since been understood by the term, *hypnotism*. He carefully put hypnotism to the test as a therapeutic and anæsthetic agent, but the results of his experiments, although of unusual interest, were such as to lead both himself and those who were familiar with them to the conclusion, that the use of hypnotism in the practice of physic could only be very restricted. While, therefore, Mr. Braid's researches yielded, on the one hand, a solid and most important addition to our physiological knowledge, on the other, they appeared to mark out so clearly the degree of utility and comparatively slight value of artificial somnambulism as a remedial or anæsthetic agent, that recourse has very rarely been

* *Cours Théorique et Pratique de Braidisme, ou Hypnotisme Nerveux, considéré dans ses rapports avec la Psychologie, la Physiologie, et la Pathologie, et dans ses Applications à la Médecine, à la Chirurgie, à la Physiologie Experimentale, à la Médecine Legale, et l'Education.* Par le Dr. J. P. Philips. Paris: Bailliere, 1860.
*Recherches sur l'Hypnotisme, ou Sommeil Nerveux, comprenant une Série d'Expériences Instituées à la Maison Municipale de Santé.* Par MM. les Docteurs Demarquay et Giraud-Teulon. Paris: Bailliere, 1860.

had to it by the English physician, and the whole question of its therapeutic value has fallen into abeyance.

In France, notwithstanding that the remedial properties of artificial somnambulism induced by *mesmerism* had oftentimes been discussed, the healing virtues of somnambulism brought about by *hypnotism* appears to have attracted little notice; indeed, the whole question of hypnotism does not seem to have excited much attention there until the termination of last year, when the subject, in its bearing upon anæsthesia, was brought before the Academy of Sciences, by MM. Velpeau and Broca, *apropos* of an experiment (the opening of an anal abscess during hypnotic insensibility in a female aged forty years), performed by Drs. Broca and Follin, at the hospital at Necker. Drs. Demarquay and Giraud-Teulon appear next to have taken up the question in the *Gazette Médicale de Paris* (December, 1859, and January, 1860), and about the same time Dr. Azam, the assistant-physician at the lunatic asylum of Bordeaux, sought to verify Mr. Braid's researches, and made known the results of his inquiry in the *Archives Générales de Médecine* (January, 1860).

A few days after the communication of Drs. Broca and Follin's case to the Academy, Dr. Guerineau, of Poictiers, transmitted to the same learned body an account of an amputation of the thigh, which he had performed while the patient was in the hypnotic state. "After the operation, which endured a minute and a-half," writes M. Guerineau, "I addressed the patient, and asked him how he felt. He replied that *he was in paradise*, and seizing hold of my hand, carried it to his lips to kiss it. He said also to a student, 'I felt (although without pain) what was done, because the thigh was cut off at the moment when you asked me if I suffered any pain.'"*

The question of hypnotism had now been fairly broached in France, and the circumstances seemed favourable for the further study of the biological and medical questions linked to artificial somnambulism. So thought Dr. Philips, an old and enthusiastic worker in the mesmeric and hypnotic field. He was fully acquainted with the importance and significance of Mr. Braid's researches, and regarding them as the clue to a vast, uncultivated field of observation, had long worked to gain for them at least the same degree of attention in France that they had received in England, but in vain. It was in an interval of this labour, partly caused by failing health, that the first communication on hypnotism was made to the Academy; but Dr. Philips shall tell his own story.

"Sundry circumstances, among others, defective health, had pre-

* *Gaz. des Hôpitaux*, Dec. 29, 1859. Quoted by Dr. Philips.

vented me pursuing this work. But a few words uttered before the Institute of France by a celebrated surgeon have realized in a few days that which the long and laborious efforts of an obscure man had failed to accomplish. I had undertaken to interest the medical world in questions which up to that time it had looked upon with prejudice and repugnance; the illustrious introducer of *hypnotism* to the Academy had conquered by his patronage the attention and interest of the entire world. A longing to place my experience at the service of the eminent men who had taken the cause in hand to which for so many years I had devoted myself, at once took me from my asylum in the fields. But a painful surprise awaited me at Paris; I was there given to understand that the champions of hypnotism had scarcely appeared when they wheeled about, and retired precipitately to their tents." (p. x.)

Such indeed was the case. "Cooled," say Drs. Demarquay and Giraud-Teulon, "by an exaggerated deception which might have been easily avoided, and regretting their prompt enthusiasm, the promoters of hypnotism hastened to bury an idol that they had eagerly sought to elevate upon an altar."

Notwithstanding, however, this serious defection, the question was not destined to rest here and prove entirely abortive. The impulse generated within the walls of the Academy had gone forth, and of the more immediate extra-academical results the works of Dr. Philips and Drs. Demarquay and Giraud-Teulon are examples.

Nothing could well be conceived to differ more the one from the other, short of entire dissidence, than the writings of these gentlemen. Dr. Philips, full of enthusiasm, stands on tip-toe upon the results of Mr. Braid's experiments, which he conceives to have been amply verified by his own researches and those of others, and he endeavours to peer far beyond the bounds of a distant horizon: Drs. Demarquay and Giraud-Teulon, full of scepticism, seek to restrict the horizon within the ken of an extremely myopic vision. Neither a too great enthusiasm nor a too great scepticism can, however, destroy the peculiar interest which belongs to hypnotism, and we shall endeavour to cull the chief facts, asserted or testified to, recounted by our authors. Once and for all, we shall not suffer ourselves to be tempted by them into the pleasant and illimitable field of theory or hypothesis. If Dr. Philips thinks that hypnotism is yet but in a rudimentary state, and that when perfected it is destined to effect eminent good in surgery, therapeutics, physiology, psychology, education, and morals, nay, even may prove the key to unlock the mysteries of the soul, the law which governs the moral and physical worlds, and make apparent the intermediate agent by means of which the two poles of life are linked together, we shall not quarrel with these truly "gigantic hopes." If it be Dr. Philips's intention to attempt to justify these hopes, we would but throw out the caution that the loftier the aim the greater the necessity for securing firmly under foot every, even the slightest step, towards its attainment. We must even put aside Dr. Philips's theoretical exposition of the phenomena of hypnotism, notwithstanding its ingenuity, and this from no disrespect to him, but from a feeling (it may be a perverse one; certainly it is an obstinate one) that hypnotism requires cultivating experimentally much more than speculatively.

If we disregard the theoretical waifs of Drs. Demarquay and Giraud-Teulon, it is because they are utterly disproportionate in magnitude with the experimental observations upon which they are based—these observations, while of interest in themselves, having no value as tests of experiments performed under entirely different conditions.

Dr. Philips objects to the term *hypnotism*. This, like the term *animal magnetism* applied to the great collateral method of inducing artificial somnambulism, he considers to be inexact. The last designation implies a disputed theory; the first conveys the erroneous idea that "the essential and constant character of the phenomena it represents is sleep." He would substitute, therefore, the word BRAIDISM for hypnotism, as (which is, indeed, now chiefly done in this country) MESMERISM for animal magnetism, "according to a rule already consecrated in the classical words *galvanism, voltaism, faradism.*"

Notwithstanding the somewhat uneuphonious character of the word *Braidism,* its adoption would certainly possess the advantage set forth by Dr. Philips, and is not inconsistent with the canons of nomenclature and terminology. Moreover, we are inclined to have recourse to it as a tribute to the memory of Mr. Braid, whose loss* we have so recently had to regret. A sufficiency of euphony may even be secured for English ears, if the word be written with a diæresis, thus, *Braïdism,* and pronounced accordingly. This is the course that we shall adopt in the subsequent portion of this article.

The chief phenomena of Braïdism are summed up by Dr. Philips in the following categories:—

1. Resolution of the voluntary muscles extended throughout the whole system, or localized in an isolated portion; catalepsy, tetanic contractions, clonic contractions and uncontrollable coordinated movements, and considerable augmentation of the muscular power.

2. Hyper-excitation or annihilation of the general sensibility. Both the hyperæsthesia and anæsthesia may extend over the whole

* Died on the 25th March last.

body, or may be circumscribed, as may be required, to a more or less restricted portion; for example, a single member, an arm, a thigh, or even to a single phalanx of the fingers. Exaltation, suppression, and perturbation of the special sensibility; illusions of the senses, the impressions received from exterior agents giving place to a sensation foreign to their properties. Thus water may be mistaken for wine, and an object of the same temperature as the air, placed in contact with the skin, may produce the sensation of red-hot iron. Finally, objective perceptions appear to take place in the sensorium, without participation of the external organs of sensation.

3. The energy of the intellectual and moral faculties are stimulated or enfeebled, and their habitual activity increased in an indeterminate degree. The taste for music, for example, the sentiment of time and tune, acquire an exquisite delicacy in the least musical organization; the most feeble memory becomes endowed all at once with an almost infallible strength, or else it is completely disordered, or it suffers from a special or partial lesion, certain names, certain letters, or certain dates being forgotten. Habitudes of thought may also become changed in a greater or less degree, from which it follows that their relative action is susceptible of change, and that consequently the type of character can undergo a veritable transformation. Thus the choleric may become placid, the humble arrogant, and the timid and pusillanimous proud and courageous.

4. The involuntary muscles are affected in a manner analogous to the voluntary; the circulation may be hurried or impeded; the activity of the secretory functions increased or diminished; in a word, all the operations of vegetative life may be more or less modified.

The Braïdic state may be induced, almost indifferently, by any means calculated to obtain fixity of attention on the part of the subject. Mr. Braid made use either of a brilliant object, held a short distance in front of the patient's eyes, or a prominent object fixed upon the forehead, or he directed the subject's attention to some point in the room, or he made use of the ordinary mesmeric method. Now, according to Dr. Philips, the Braïdic operation has two distinct stages. In the first, the object is to "develop a preparatory modification of vitality, a modification which is often latent, and of which the effect is to fit the organization to undergo the determining and specific action which constitutes the second stage. This preliminary modification he designates the *hypotaxic state* (*état hypotaxique*), and the act of producing it he terms *hypotaxis* (*hypotaxie,—υπόταξις* preparation, to undergo). In the second stage of Braïdisation, the special functional modifications are sought to be developed. "The impression employed for this end is a *mental impression,* that is to say, a suggested idea. And as this idea becomes thus the determining agent of the functional modifications to be provoked, the general application of the proceedings which constitute the second period of the *Braïdic* operation ought to bear the name *ideoplastic* (ideoplastie).

Dr. Philips estimates that about one out of every thirty-five or forty individuals met with will be found predisposed to succumb to Braïdisation. The persons who are thus liable are generally of a nervous temperament; but instances are met with of individuals who appear to have enjoyed habitually good health, and never to have suffered from any nervous disorder. Outside this category of persons who are highly susceptible, the aptitude to submit to the action of Braïdism varies considerably in degree. This aptitude, moreover, is liable to vary in the same individual at different times; and even those who have exhibited the least disposition to yield to the operation have acquired the aptitude after a course of daily Braïdisations.

The bilio-nervous temperament, Dr. Philips thinks, seems to predispose more than any other to Braïdism. But the conclusion may be illusory, for he remarks that, if the majority of the individuals found liable to the hypotaxic state manifest signs of a bilio-nervous temperament, persons of this temperament take the most interest in his demonstrations, show the greatest desire to submit themselves to experiment, and form invariably the greater portion of his experimental *personnel.*

The conditions of *character* would appear, however, according to the same authority, to be clearer and more determined than those of *temperament.*

"The predominance of elevated mental emotions above the grosser or personal instincts, a serious humour, and especially a disposition to confidence and faith, are favourable moral conditions; egotistic penchants, an exaggerated tendency to scepticism and criticism, and lightness of spirit, constitute refractory dispositions.

"He who, either from the mobility of an over-excited imagination, or from the invincible energy of his reflexion, or from debility of his intellectual faculties, or from want of a sufficient command over the operations of his mind, is incapable of fixing his thought for a given time upon an object, and holding it concentrated on a simple and limited circle of ideas, is eminently unfitted for the hypotaxic state. In virtue of the law of contact of extremes, idiots and the most robust and active understandings are found united in this category of incompatibility.

"We have again and again observed that persons habitually impressionable, and even those who were so in the highest degree, ceased to be so when they were dominated by a vivid prepossession." (p. 42.)

From ten to twenty-five or thirty years is the somewhat wide period, according to Dr. Philips, when the aptitude to the hypotaxic state attains its apogee; but as to the liability of sex, his observations do not permit him to pronounce positively, he having chiefly experimented on males. A full stomach has a fatal influence upon the induction of the hypotaxic state.

One of the least satisfactory portions of Dr. Philips's work is that devoted to the illustration of the presumed therapeutical value of Braïdism. To make this manifest, he has recourse chiefly to the recorded experience of other writers. This is most scanty at the best, and we should have been better pleased to have seen a careful account of Dr. Philips's own observations on the therapeutic virtues of the hypotaxic state. The want is all the more apparent considering that his treatise arose out of a discussion in which the medical rather than the physiological properties of hypnotism were in question. If it were not for this defect, and for the dubious theoretical matter it contains, Dr. Philips's work would have proved a most welcome addition to medico-psychological literature; but as it is, the book must be characterized as a useful and interesting manual of hypnotism or Braïdism.

Drs. Demarquay and Giraud-Teulon put hypnotism to the test upon eighteen subjects, in the Maison Municipale de Santé. Fifteen of the subjects were women, and three men; and eleven of the former were invalids. Of these, eight were suffering from serious affections of the reproductive organs (affections graves de l'appareil genital). The males, the four healthy females, and one of the patients suffering from uterine cancer, were not affected in the least degree by the hypnotic process adopted; the remaining subjects were affected in different degrees. The number of experiments made amounted to forty; the method in which they were made was as follows:—

In the earlier experiments the mirror of an ophthalmoscope, and in the latter ones a small bright metal sphere, was placed in such a position as to induce slight straining and convergence of the eyes when they were both fixed upon the object. Further, looking upon Mr. Braid's observations as a "somewhat crude *mélange* of the psychical and purely physiological element," Drs. Demarquay and Giraud-Teulon sought to set aside even the suspicion of a foreign psychical or moral influence disturbing their experiments, hence, "placing their subjects in exclusive relation with the sphere or the mirror, the object of their regard, shunning to attract their attention, leaving them, for the most part, in ignorance of the end essayed, we," they assert, "freed ourselves from all action which was not exclusively physiological and anti-biological" (p. 15); that is to say, these gentlemen reduced the psychical element of the experiment to the lowest degree possible. Indeed, they conceived that they had so completely effected this, that they came to the ultimate conclusion that the key to all verified hypnotic phenomena, and sequentially to the most marvellous of the superstitions of the Middle Ages was, the *fixity of regard* bestowed by the subjects of experiment upon the small bright object set before them. "*Fixity of regard!*" write these experimentalists, in their section concerning "Hypnotism dans les Temps Historiques; demonomania, possessions diabolique," &c., "that is the secret common to all these processes, differing solely in its value and efficacy, developing more or less rapidly the anticipated effects (effects attendu), according to the time employed and the subjects submitted to experiment. Possession, demonomania, magnetism, cataleptic ecstasy, somnambulism, hypnotism, are one and the same state, manifesting more or less religious enthusiasm (l'exaltation mentale religieuse) with the series of special disorders which may follow these monomanias. Such is the scientific tableau traced by modern experiment upon the canvas bequeathed by history" (p. 44). Such is the magnificent culmination of the train of argument which Drs. Demarquay and Giraud-Teulon develope out of their experiments, and yet they hold up Mr. Braid to animadversion for having mixed up the psychical element with his experiments, assert this to have been an unpardonable error against the rules of physiological experiment, and reject as improbable and wanting in verification the whole of what may be termed the higher phenomena of hypnotism educed by him !

Now, let us endeavour to ascertain the actual value of Drs. Demarquay and Giraud-Teulon's opinion upon a question of experimental physiology. Their sensitive subjects were all invalids, and with one exception suffering from serious diseases; Mr. Braid's were healthy individuals, chiefly selected at random from mixed audiences, or picked up incidentally. Dr. Philips's description of the temperament and character of his subjects will apply also to Mr. Braid's subjects. Drs. Demarquay and Giraud-Teulon eschewed all psychical influence ; Mr. Braid attributed the results of hypnotism chiefly to psychical influence, and it was this opinion, accurately reasoned upon, that led him to institute those experiments by which he was enabled to develope and show the nature of the extraordinary phenomena depending upon exaltation of the smell and of the muscular sense, and upon suggestions derived from the last-named sense and from auditive impressions. The same reasoning also gave him the clue to the so-called odylic phenomena, and led him at once to demonstrate experimentally their true character. In short, it was by his skill in playing upon the mind of his subjects that Mr. Braid induced the higher phenomena of artificial somnambulism, and those equally interesting phenomena which may be evoked in certain individuals not hypnotized, and which led Baron Reichenbach astray.

Thus it is apparent that neither in the class of subjects submitted to observation, nor in the method of conducting the observations were the experiments of Drs. Demarquay and Giraud-Teulon and those of Mr. Braid at all similar; therefore, in endeavouring to test the value of Mr. Braid's experiments by their own, Drs. Demarquay and Giraud-Teulon have sinned against the merest rudiments of experimental observation; and their conclusions are of no value whatever in their bearing upon Mr. Braid's experiments, except in so far as the experiments of the former gentleman approximate in character to those of the latter gentleman, in which case the results tally to a nicety.

The observations of Drs. Demarquay and Giraud-Teulon betray a very imperfect acquaintance with Mr. Braid's writings, and it is to this alone that we can attribute the singular errors committed by these gentlemen in their criticisms upon the last-named gentleman's experiments. Moreover, Drs. Demarquay and Giraud-Teulon seem to be ignorant of the fact, that the chief of Mr. Braid's experiments had been verified by no less distinguished a physiologist than Dr. Carpenter.

It might at least have been expected that Drs. Demarquay and Giraud-Teulon would have tested the value of their own theory, that to "*fixity of regard*" solely was to be attributed the hypnotic phenomena they observed. Had they instructed their subjects to fix their eyes upon the glittering object placed in the required position, and contrived to direct their attention without disturbing the visual organs, we fancy they would quickly have found that a *fixity of attention* as well as a fixity of regard upon the object was required to mature the experiment; that, in short, a psychical element was always present in the experiment, and was essential to its success.

Be this as it may, however, the positive results which Drs. Demarquay and Giraud-Teulon derived from their experiments are not without interest. These were, (1) an oscillatory state of the pupils during the production of hypnotism, although this was not constantly observed; (2) anæsthesia, occasionally marked, most frequently feeble, often absent; (3) lowering of the pulse, but less regularly than diminution of the respiratory rhythm; (4) general hyperæsthesia in one instance.

Of the anæsthetic influence of the hypnotic state upon patients actually suffering pain, Drs. Demarquay and Giraud-Teulon write:—

"Nearly all the patients in whom we have succeeded in inducing the nervous sleep suffered from grave affections of the genital apparatus: we do not say all. Now in all these cases it is a fact which has been reproduced whenever hypnotism has been determined, that very acute uterine pains, which tormented these unfortunates day and night, and of which they complained bitterly before the nervous sleep, were checked and suspended during this particular state of their nervous system, and long afterwards; twenty hours of perfect comfort being the mean duration of the solacement; and it was so real, so incontestable, so patent, that the patients demanded to be hypnotized immediately upon being visited. One young demoiselle who suffered cruelly from neuralgic pains in the pelvis (the consequence of a violent contusion with fracture) and which had not been relieved either by opium, or by chloroform used throughout an entire night, was calmed as by enchantment, and for a period of twenty hours, by hypnotism; and this two days in succession.

"These facts were reproduced with sufficient constancy to fix the attention and merit notice. They may give rise to new indications for the employment of this singular process, and open out a new path for the treatment of neuralgic affections. It is to be understood, however, as far as we are concerned, that this aptitude is limited to the special circumstances in which we have observed it, that is to say, neuralgic affections bound to certain special states of the organism allied to hysteria." (p. 20.)

These results, it will be seen, add nothing to what had already been made manifest by Mr. Braid, and the recent interest exhibited for hypnotism in France has not yielded hitherto one new fact, physiological or therapeutical, in hypnotic science. Mr. Braid's researches opened out a wide and highly interesting field of experimental research, which doubtless admits of further and very profitable cultivation. In the meantime, the question raised by Dr. Phillips awaits decision, whether in future the term BRAIDISM shall be substituted for HYPNOTISM?

---

